# The Therapeutics of Infancy and Childhood

**Published:** 1894-08

**Authors:** N. G. Gewinner

**Affiliations:** Macon, Ga.


### THE THERAPEUTICS OF INFANCY AND CHILDHOOD.*

By N. G. Gewinner,

Macon, Ga.

There is not a subject of more vital importance to the general practitioner, than that of the "Therapeutics of Infancy and Childhood." A physician may be ever so accurate in his diagnosis, but if he does not give the right remedy or the proper dose thereof, his patient will not be benefited.

Apart from the general principles which effect changes in therapeutics, such as climate, sex, custom, idiosyncrasy and physiological status, infancy and childhood require special study. Infancy extends from birth to about the age of three years, or till the completion of first dentition. Childhood from that time to puberty, or the age of fourteen years.

There is scarcely an organ which behaves in the young as it does in later life. They are more delicately organized, and contain a

*Read before the Macon Medical Society, April 3, 1894.

larger proportion of water and are more easily injured. The bones contain less earthy matter, animal matter predominating. The brain is being rapidly developed, much more so than any other organ. The arterial pressure is not so great. The digestive organs of infants present many peculiarities. The digestive glands are but gradually prepared for their functions. The salivary glands are only partially active. The secretions of the stomach contain more lactic than muriatic acid. The stomach is less elongated and occupies a more vertical position than in the adult, hence vomiting is more frequent, more readily produced, and causes less disturbance to the general system. The liver is much larger in proportion to the size of the body than it is in adult life. All of these differences must be taken into consideration in treating infants and children, and necessitates changes in remedies and doses.

Bartholow says, "Many highly approved methods of treatment, which, in the case of adults, are much employed, cannot be in children because of difference in age, insusceptibility to impressions, tenderness of the tissues and rapidity of the nutritive processes. On the other hand, in consequence of the great activity of the tissue metamorphoses, some modes of procuring medicinal action, not effective in the cases of adults, become highly so in children."

In prescribing for children we should bear the following in mind:

1st. The use of such medicines as can be given in small doses and produce the desired effect.

2d. Their administration in such form as will render them most palatable.

Children are greatly unlike adults in regard to medicine. Many adults do not think that their physician is doing anything for them unless they have a dose to take every hour or two. Children generally, instinctively I might say, fight strenuously against even palatable mixtures; therefore it is well to give them what will produce the effect desired in as few doses as possible.

I have seen the good effects of many doses of medicine administered to children not only counterbalanced, but, as I believed, even made a positive injury to the child through the struggles which resulted from attempting to administer the drug.

It, therefore, follows that the dose of medicine administered to such children should be such as to necessitate as little repetition as possible. Medicines may be administered, or rather children may be treated by mouth, by rectum, by the skin, embracing inunctions, baths, hypodermatic injections, endermatic applications, and counter-irritation. They may also be treated by inhalations. In administering medicines by mouth, we should always remember that dosage cannot for a given drug be set at one categorical figure, and that even with given conditions, dosage can never be estimated with any approach to precision. This is especially true of a child who struggles very much against taking a medicine. He may have one-third of the dose in his mouth, and two-thirds on his face and clothing.

Various rules have been formulated for approximating the dose of medicines required at various ages.

Dr. E. H. Clark, of Boston, proposed the following: The weight of the adult being fixed at one hundred and fifty pounds as the standard, this must be regarded as unity or one. Accordingly, the dose for the child will have the same relation to one or unity, as its weight has to one hundred and fifty pounds. If the weight of the child be divided by one hundred and fifty, the resulting fraction is the proper dose. Thus for a child of eighteen months, weighing twenty pounds, the dose would be $\frac{20}{150} = \frac{2}{15}$ or about one-eighth of the adult dose.

Dr. Conley, of Louisville, Ky., gives us the following: Take the next ensuing birthday and divide the number by 24. If the child's next birthday is 4 and this is divided by 24 the result is $\frac{4}{24} = \frac{1}{6}$. Then a child of four years of age would require one-sixth of an adult dose.

The method of Dr. Young, which has long been known, is to divide the age of the child by the age plus twelve. Thus, if the age of the child is four divide this by four plus twelve (sixteen), and we get one-fourth as the proper dose.

These rules cannot be of mathematical accuracy, neither can they be arbitrary. The therapeutics of infancy and childhood are by no means so similar to those of adults that the simple proportionate reduction of dose according to a given rule will answer. Some remedies are much better borne in children in proportionately larger doses than they are by adults. Other remedies are not so well borne. Opium and its preparations must be handled with care. Children, as a rule, are very susceptible to its effects and relatively smaller doses must be given, or, better still, do not give it at all unless nothing else will answer, then watch it closely. Chlorate of potassium demands great care. Carbolic acid becomes poisonous to the very young in small doses and even externally. Among the antifebriles cold is tolerated less, quinine more in proportion than in the adult, so also are antipyrin, antifebrin and phenacetin. Heart stimulants are also borne in relatively larger dose, thus digitalis, strophanthus, sparteine and strychnine. Caffeine must be used with caution and only when there is positively no cerebral complications of a congestive or inflammatory nature. Preparations of arsenic are well borne, and may be kept up for rather longer time. Lobelia and belladonna are borne in rather large doses, and hyoscyamus can be given in much larger doses proportionately in spasmodic conditions of the bladder than in advanced age. Mercurials generally can be given in rather larger doses. Mercury does not salivate children so readily but may produce rapid anemia. Many of the powerful medicines are required in smaller doses, others in larger doses. I never prescribe chloral hydrate without exercising great care and watchfulness. The bromides are generally well borne by children, but their continued use is more apt to produce the characteristic rash than in adults.

Two-thirds of the ailments of infancy and childhood being of digestive or alimentary origin, mild cathartics are frequently required and do a great deal of good. This fact is so well recognized by the laity, that children are too frequently brought to death's door by the indiscriminate purging with castor oil, rhubarb, calomel and patent syrups of hocus-pocus, etc. Children bear cathartics well, but their organs are so sensitive that many, very many, have been brought to untimely graves by the too persistent use of cathartics and anthelmintics.

There is one remedy, or rather I should say prophylaxis, that always acts well with all children, and which, if properly carried out would do much to lessen the fearful mortality of infancy and childhood and would literally bring about, as far as the little ones are concerned, the throwing of medicine to the dogs. I allude to that all important part of therapeutics—diet. Nearly all of the digestive and alimentary diseases attributed to the teeth, hot weather, colds, etc., would be prevented by proper feeding and plenty of pure water; food that would be nourishing and easily digested, and water that would flush out the *materies-morbi* of the little body and keep it in health.

The treatment of children by the rectum is very important and often does what cannot be done by a dose of medicine administered the usual way. Rectal injections of warm water in sweeping out scybala and relieving obstinate constipation, or washing out with medicated enemas that annoying parasite in children, the ascaris vermicularis; in relieving the tenesmus in dysentery; in administering drugs, or even food, when they cannot be taken by mouth, and in many other ways, the treatment per rectum is of the greatest importance. Children may also be treated very effectually through the skin. Inunctions are of great value. Animal fat, well rubbed in, go far to sustaining and building up a feeble child. Inunctions of oil are of great benefit in reducing the fever by allaying the itching in the exanthemata. Oleates of quinia and mercury are sometimes very effective when rubbed over the large glands just after a warm bath. Digitalis leaves made into a pad, moistened with vinegar and applied over the stomach will soon give the characteristic effects of the drug. Baths, hot and cold, are of great value; hot baths in bringing about reaction from a chill or collapse, or in congestion. The cold bath, the greatest of all antipyretics, in reducing temperature. The cold sponge bath as a tonic. Medicinal baths have also been used but not with so pronounced benefit. Hypodermatic, or, as we generally term it, hypodermic, injections are not as freely used in treating children as they are with adults.

The reasons of this are the pain in giving the injection, the danger of abscess and the difficulty in grading the dose. They should never be given to children under three years of age, except in extreme cases. The dose should not be more than one-third of the proportionate dose, and in using preparations of opium should even be less.

About the same line of drugs may be used in this manner with children as with adults. I will mention one particularly which is highly recommended in the uremic convulsions of children—that is, pilocarpine. It should be used with the greatest caution, and only the smallest dose until satisfied that the child has no idiosyncrasy which would contraindicate its use.

Endermatic application by first blistering the skin and then applying the medicament, is too painful a process to be used except in extreme cases where no other hope exists. It can be done by brushing the part with pure carbolic acid, or by moistening the inside of a watch glass with strong ammonia and holding tightly over part to exclude the air. After a blister has been formed remove the outer skin and apply the medicine, either as an oleate or in a powder sprinkled over the surface. Quinine can be administered in this way with good effect Counter-irritation, not carried to the point of blistering, often gives our little patient prompt relief. The mustard plaster or some stimulating liniment quickly relieves pain in many cases. Mustard plasters should, however, be watched, as on the tender skin of children they will readily produce a blister, and a blister from mustard is one of the most painful. After removing the plaster the part should be well washed with warm water to remove any adherent particles of mustard, and if much redness and burning remains some soothing oil should be applied. Blistering with flies should never be resorted to with children except as a last resort and in very grave cases.

Other applications which are not, strictly speaking, counter-irritants but yet act to some extent in that way, are poultices. Who of us cannot recall our boyhood days, the mess of green apples, that fearful pain and the relief that the hot poultices of mush afforded. And how hot they were. Yea, my abdomen feels warm even now thinking of those hot poultices. In truth I have sometimes thought that the pain from the great heat outside made me oblivious of the inside disturbance.

Poultices, whether of meal, bran, flaxseed, hops or other material, are of great benefit when properly made and applied so as to retain the heat and moisture. Poultices of flaxseed meal and mustard are of especial benefit in the bronchial and lung troubles of children.

Of inhalations the most important and frequently used in disease of children are ether and chloroform to produce anesthesia. Chloroform in the convulsions of children sometimes has a happy effect. Inhalations of medicated vapor are used in throat and bronchial troubles. With great benefit in dissolving the membrane in croup and dipbtheria. Sprays and douches all play their part in the therapeutics of children.

Electricity is also a valuable agency for good in treating children, especielly useful in reducing strangulated hernia and sometimes in relieving impaction and intussusception.

---

### OLIVE OIL IN OBSTRUCTIVE JAUNDICE.

Oliver (*Lancet*, No. 3658, 1893) reports two cases of simple obstructive jaundice successfully treated with olive oil. The first patient complained of sudden attacks of severe pain in the upper part of the abdomen, which of late had become more frequent and severe, and had occasionally been attended with vomiting and followed by jaundice. On account of increasing debility the writer finally prescribed olive oil, beginning with one tablespoonful in milk daily and gradually increasing the amount to six tablespoonfuls. With the exception of a slight attack of colic on the second or third day after the treatment with the oil was instituted, the patient had no further pain and no return of jaundice, and is now in better health than he has been for the last five years. Treatment was continued for several weeks after the disappearance of the symptoms, and in addition to the oil he was given two grains of calomel twice a week, and a few drops of extract of cascara sagrada every evening.

The second case, a woman forty-eight, had been deeply jaundiced for ten months. The abdomen was retracted and the liver enlarged. She was very feeble and her mind was depressed. As all other remedies had proved futile she was given daily two tablespoonfuls of olive oil in warm milk. Within three weeks the jaundice disappeared, the stools became normal and there was a remarkable improvement in the general condition. The author is unable to give any explanation of the action of the oil in these cases.—*University Medical Magazine.*

